# Impact of Protein and Carbohydrate Supplementation on Musculoskeletal Injuries in Army Initial Entry Training Soldiers

**DOI:** 10.3390/nu10121938

**Published:** 2018-12-06

**Authors:** Kaitlin D. McGinnis, Jeremy S. McAdam, Christopher M. Lockwood, Kaelin C. Young, Michael D. Roberts, JoEllen M. Sefton

**Affiliations:** 1 Warrior Research Center, School of Kinesiology, Auburn University, 301 Wire Road, Auburn, AL 36849, USA; kdm0031@auburn.edu (K.D.M.); jsm0039@auburn.edu (J.S.M.); 2 UAB Center for Exercise Medicine, University of Alabama Birmingham, Birmingham, AL 35294, USA; 3 Lockwood LLC, Draper, UT 84020, USA; chris@drchrislockwood.com; 4 Department of Cell Biology and Physiology, Edward Via College of Osteopathic Medicine (Auburn Campus), Auburn, AL 36849, USA; kyoung@auburn.vcom.edu; 5 Molecular and Applied Sciences Laboratory, School of Kinesiology, Auburn University, Auburn, AL 36849, USA; mdr0024@auburn.edu

**Keywords:** musculoskeletal injuries, initial entry training, basic combat training, whey protein, energy intake, military medicine

## Abstract

This project investigated whey protein and/or carbohydrate supplementation effects on musculoskeletal injury (MSI) outcomes. Four groups of Initial Entry Training soldiers consumed either: (1) one protein (38.6 g, 293 kcal); (2) one carbohydrate (63.4 g, 291 kcal); (3) two protein (77.2 g, 586 kcal); or (4) two carbohydrate servings/day (126.8 g, 582 kcal) after physical training and before bed, or before bed only. Odds Ratio, Chi-square and Wilcoxon ranked-sum test compared supplementation/no supplementation, number of servings, and protein/carbohydrate for MSI and limited/missed duty rates and limited/missed training days. Non-matched pairs group averages were compared to 2015/2016 historical data. Non-supplemented soldiers were approximately 5× more likely to sustain a MSI (*χ*2 = 58.48, *p* < 0.001) and 4× more likely to miss training (*χ*2 = 9.73, *p* = 0.003) compared to two servings. Non-supplemented soldiers missed five additional training days compared to two servings (*W* = 6059.5, *p* = 0.02). Soldiers consuming one serving were approximately 3× more likely to sustain a MSI than two servings (*χ*2 = 9.55, *p* = 0.002). There was no difference in limited/missed duty rates or limited/missed training days between consuming one or two servings. There was no difference between consuming one serving versus no supplementation or protein versus carbohydrate supplementation for any outcome variable. Soldiers consuming 2 servings/day of protein or carbohydrate had lower MSI rates, limited/missed duty rates, and limited/ missed training days compared to non-supplemented soldiers.

## 1. Introduction

Initial Entry Training (IET) is a physically and cognitively demanding course designed to transform civilian volunteers into United States (US) Army soldiers. The course trains and assesses soldiers on physical and mental tasks centering around physical training, physically demanding occupational tasks (e.g., ruck marching, land navigation) and classroom-based knowledge. Military recruits are representative of the US population, with increasingly poor physical fitness and sedentary rates [1]. The physically demanding training is difficult for unfit recruits to successfully navigate resulting in lower levels of IET completion [1,2]. Recruits who cannot complete IET cost the US Army approximately $300 million dollars each year [3]. The IET attrition rate is 6–14%, largely due to musculoskeletal injury (MSI) [3]. Approximately 25% of male recruits and 50% of female recruits sustain a MSI during IET [2], which accounts for 80% of all medical discharges during the first year of service [4]. High MSI rates lead to high medical and financial costs, missed training days, lower numbers of deployable personnel, and physical and emotional damage to the individual soldier. Investigations into high MSI rates during IET have indicated that age, initial physical fitness, body composition, smoking, and gender are possible contributing factors [5,6]. However, little research has been completed on the impact of nutrition on MSI within the US Army IET. Proper nutrition likely plays an important role in performance of tactical athletes. Nutrition has been shown to impact fatigue [7], muscle recovery [7], and cognitive function [8,9]. Thus, it is important that tactical athletes match calorie intake to energy expenditure during IET. Failure to do so can reduce physical performance and may contribute to MSI [10]. However, research has indicated that IET soldiers complete training in a significant caloric deficit and are specifically deficient in protein and carbohydrates [11,12].

Protein, carbohydrate, and overall caloric intake could be improved through changes in diet or through supplementation. Protein supplementation has been shown to have a positive effect on muscle protein synthesis [13,14,15], lean body mass [16,17], biomarkers of recovery [18], performance [18], and osteoblast activity [11,19]. Carbohydrates are known to be important for energy metabolism and glycogen re-synthesis following exercise [20]. To date, little data exists with regard to examining the physiological effects of protein or carbohydrate supplementation in IET. One 54 day study [21] examined the effects of protein supplementation (10 g) in IET marines. These authors reported protein supplementation reduced medical visits for the treatment of muscle or joint pain relative to marines supplemented with carbohydrate or no supplementation [21]. There was also a significant decrease in muscle soreness in marines that supplemented with protein [21].

Initial research indicates that IET soldiers are not meeting the requirements for calories, protein or carbohydrate intake for active individuals [12,13]. However, no research to date has assessed the effect of nutrition on MSI rates in IET. Therefore, the purpose of the current study was to determine if protein and/or carbohydrate supplementation in IET soldiers influenced MSI rates, the rate of MSIs requiring limited/missed training days (limited/missed duty rates), or total number of limited/missed training days (limited/missed training days).

## 2. Materials and Methods

A double-blind control study evaluated the risk of MSI, limited/missed duty rates, and the number of limited/missed training days in IET soldiers supplementing with whey protein or carbohydrate. Possible participants were informed of study procedures by a member of the research team during a company brief. Participants were required to be at least 18 years of age, have no prior supplementation (whey protein or ergogenic aids) within the last 6 months, no current MSI, and no allergy to milk or whey protein. Volunteers completed the informed consent. This study was conducted in accordance with the Declaration of Helsinki and protocol was approved by the Auburn University Institutional Review Board (IRB), and the Director, Research & Analysis Directorate Army Center.

Two IET companies participated in this study. One completed Basic Combat Training (BCT; 10 total training weeks), and one completed One Station Unit Training (OSUT); consisting of Advanced Individual Training in addition to Basic Combat Training (15 total training weeks). Protein or carbohydrate supplementation groups were assigned by platoon (2 platoons-carbohydrate supplementation/company, 2 platoons-protein supplementation/company) so all participants in the same platoon consumed the same supplement. The goal was to increase compliance and simplify daily administration of supplements by drill sergeants. Soldiers consumed either one serving/day of protein, one serving/day of carbohydrate, two servings/day of protein, or two servings/day of carbohydrate in liquid-shake form. Supplementation began at the end of week one of IET and concluded at the end of IET. Therefore, participants in BCT supplemented for 9-weeks and those in OSUT supplemented for 14-weeks. To provide a non-supplemented control population historical injury data was collected for IET soldiers completing training in 2015–2016, in the same training units and at the same time of year as the supplemented soldiers. Supplements were manufactured, individually packaged and blinded by JW Nutritional, LLC (Allen, TX, USA). JW Nutritional, LLC and C.M.L. (Lockwood, LLC; Draper, UT, USA) formulated match-taste supplements. Researchers and participants were blinded to packet contents until after data analysis. Covance Laboratories, Inc. (Madison, WI, USA) assessed the nutritional profile and amino acid content of both supplements and verified identity, purity, potency, and packets composition. One protein provided 293 total kcals consisting of 38.6 g of protein (Power Crunch^®^ ProtoWhey^®^ (BioNutritional Research Group; Irvine, CA, USA) as agglomerated, partially hydrolyzed (12.5% degree of hydrolysis) 80% whey protein concentrate (Hilmar^®^ 8360; Hilmar Ingredients, Hilmar, CA, USA)), 19.0 g carbohydrates, 7.5 g fat, and 20.1 g and 9.5 g of essential and branched chain amino acids. One carbohydrate serving provided 291 total kcals, 0.5 g protein, 63.4 g carbohydrates, 3.9 g of fat, and 0.1 g and 0.0 g essential and branched chain amino acids. Supplements were provided in single serving packets blinded for contents to the study staff, drill sergeants and soldiers. Soldiers consumed their supplement mixed with water prior to bed (1 serving/day) or after morning physical training (PT) and prior to bed (2 servings/day).

Dependent variables included number of MSIs, limited/missed duty rates, and limited/missed training days for each participant throughout training. A participant was determined to have a MSI if a Certified Athletic Trainer at the battalion or another member of the medical staff indicated they had sustained an MSI (MSIs were not self-reported). MSIs that were pre-existing prior to arrival at IET were excluded from the analysis (1 injury). Additionally, MSIs that occurred prior to supplementation were excluded from comparisons between types of supplementation (11 injuries). Limited/missed duty rate was the percentage of MSIs that required missed or limited training days. Limited/missed training days were counted as the number of missed/limited training days from the time soldiers were put on limited duty (began limited or no training) until they were returned to full duty. Nutritional data was not obtained from the historical cohort, thus we did not include total dietary intake data in our analyses.

Injury data was collected by the battalion Certified Athletic Trainers using a customized injury data collection sheet (Microsoft Excel, Microsoft Corporation, Washington, DC, USA). Historical injury data from 2015–2016 collected from the same Certified Athletic Training teams, completing the same training, in the same battalions, and during the same training months was used as a non-supplemented comparison.

### Statistical Analysis

Analyses were completed using Microsoft Excel (Microsoft Excel, Professional Plus 2016, Microsoft Corporation, Washington, DC, USA), R statistical software [22] and R Studio [23]. Odds ratios and Chi squared tests were calculated to determine differences in risk of MSI and/or risk of limited/missed duty between groups. Normality testing of residuals (Shapiro-Wilks and Kolmogorov-Smirnov tests) indicated groups were not normally distributed on number of training days missed. Therefore, a Wilcoxon’s ranked-sum test (W) was used to compare limited/missed training days between groups. To account for differences in the number of training days and training activities, OSUT historical data was compared to OSUT supplemented data and BCT historical data was compared to BCT supplement data. A significance value of *p* = 0.05 was set a priori.

## 3. Results

Injury data was collected from 2175 male soldiers completing IET at the Maneuver Center of Excellence; Fort Benning, GA. Demographics for soldiers consuming supplementation are located in Table 1. Demographics were not available for the historical 2015/2016 data. Companies selected for this study only trained male soldiers at the time of the study.

### 3.1. MSI Rates

**Figure 1:**
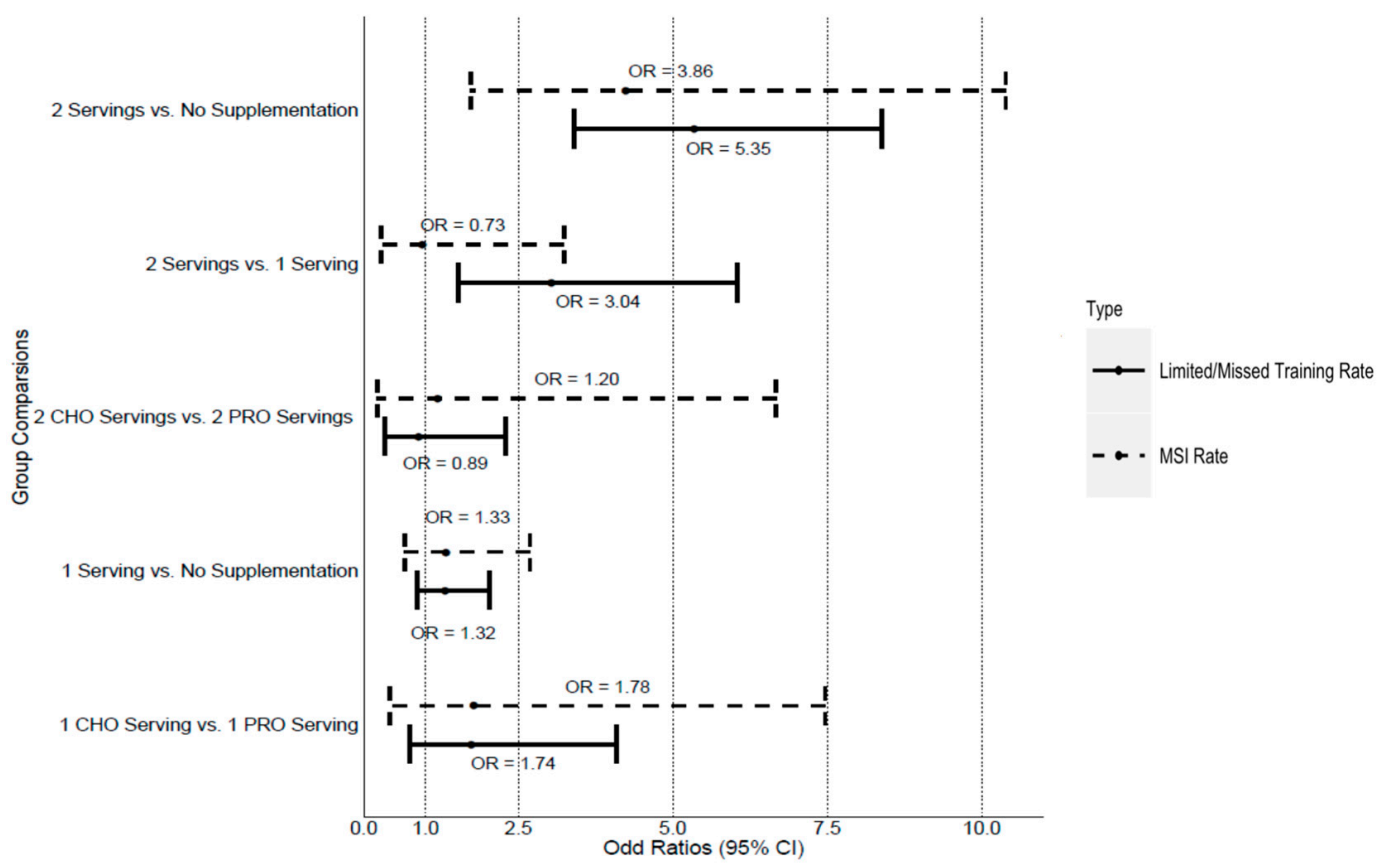
Odds ratio of MSI rates and limited/missed duty rate between groups. Abbreviations: Protein (PRO), Carbohydrate (CHO), Odds Ratio (OR).

**Figure 2:**
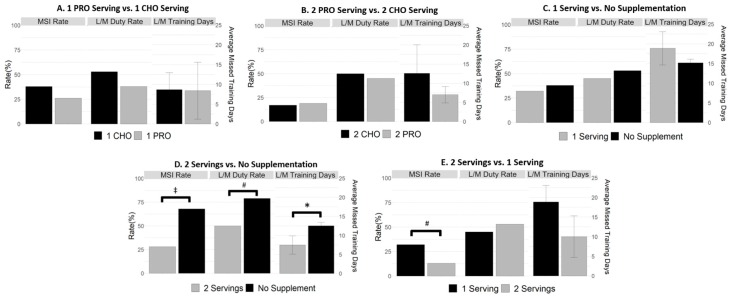
MSI Rates, Limited/Missed Duty Rates, Limited/Missed Training Days (**A**–**E**) Abbreviations: Protein (PRO), Carbohydrate (CHO), Limited/Missed Duty Rates (L/M Duty Rates), Limited/Missed Training Days (L/M Training Days), * *p* < 0.05, # *p* < 0.01, ^‡^ *p* < 0.001.

Table 2 provides a summary of MSI and limited/missed duty rates, Figure 1 provides odds ratios of MSI rates and limited/missed duty rate between groups, and Figure 2 provides MSI rates, limited/missed duty rates, and average limited/missed training days missed between groups. There was no significant difference in MSI rates between protein or carbohydrate supplementation for one serving/day (*χ*2 = 1.15, *p* = 0.28) or two supplementation servings/day (*χ*2 = 0, *p* = 1.00). There was also no significant difference in MSI rates for soldiers who consumed one serving/day (regardless of supplement type) and soldiers that were not supplemented (*χ*2 = 1.33, *p* = 0.25). There was a significant decrease in MSI rates for soldiers consuming two servings/day regardless of supplement type, compared to soldiers that were not supplemented (*χ*2 = 58.48, *p* < 0.001). Soldiers that were not supplemented were 5.35 times more likely to be injured than soldiers consuming two servings/day (regardless of supplement type). Soldiers consuming two servings/day were also significantly less likely to be injured than soldiers consuming one serving/day (regardless of supplement type) throughout IET (*χ*2 = 9.55, *p* = 0.002). Soldiers who consumed one serving/day were 3.04 times more likely to be injured than those who consumed two servings/day (regardless of supplement type).

### 3.2. Limited/Missed Duty Rates

There was no significant difference in risk of limited/missed duty between protein or carbohydrate supplementation for one serving (*χ*2 = 0.18, *p* = 0.67) or two servings/day (*χ*2 = 0, *p* = 1.00). There was also no difference in limited/missed duty rates between soldiers consuming one serving/day (regardless of supplement type) and soldiers that were not supplemented (*χ*2 = 0.067, *p* = 0.80). There was a significant decrease in limited/missed duty rates for soldiers consuming two servings/day (regardless of supplement type) compared to soldiers who were not supplemented (*χ*2 = 3.86, *p* = 0.003). Soldiers who were not supplemented were 3.96 times more likely to sustain an injury that required a limited/missed training than soldiers consuming two servings/day (regardless of supplement type). No differences in limited/missed duty rates were observed between soldiers consuming two servings/day and soldiers consuming one serving/day, regardless of supplement type (*χ*2 = 0.04, *p* = 0.85).

### 3.3. Limited/Missed Training Days

There was no significant difference in limited/missed training days for soldiers supplemented with one protein or carbohydrate serving/day (*W* = 150.5, *p* = 0.49) or two servings/day (*W* = 12, *p* = 1.00). There was also no difference in limited/missed training days for soldiers consuming one serving/day (regardless of supplement type) and soldiers that were not supplemented (*W* = 1895, *p* = 0.35). There was a significant decrease in days limited/missed for soldiers consuming two servings/day (regardless of supplement type) compared to soldiers that were not supplemented (*W* = 6059.5, *p* = 0.02). Specifically, soldiers that were not supplemented missed an average of 5.05 additional days of training compared to those consuming two servings/day (regardless of supplement type). There was no difference in missed training days for soldiers consuming two servings/day compared to soldiers consuming one serving/day, regardless of supplement type (*W* = 1908.5, *p* = 0.40).

## 4. Discussion

The purpose of this study was to determine if consuming one or two servings/day of supplemental protein or carbohydrate would decrease the number of MSIs, limited/missed duty rates, or limited/missed training days in IET soldiers. Results indicate that consuming two servings/day throughout IET, regardless of supplement type, had a positive impact on MSI risk, limited/missed duty rates and limited/missed training days compared to non-supplemented historical injury data in the same training unit. Soldiers completing IET without supplementation were five times more likely to be injured than soldiers consuming two servings/day (regardless of supplement type). Soldiers consuming one serving/day were three times more likely to be injured than soldiers consuming two servings/day (regardless of supplement type). Additionally, non-supplemented injured soldiers were four times more likely to be put on limited/missed duty. On average, non-supplemented injured soldiers required five additional missed training days before full return to duty; amounting to 470 extra missed training days/cycle compared to soldiers consuming two servings/day (regardless of supplement type). The average cost for a soldier to complete IET was $57,500 in 2005 [4]. Using these costs, a decrease of 470 missed training days/cycle would amount to a savings of $257,380/cycle [4]. Differences in MSI rates, limited/missed duty rates and limited/missed training days were independent of supplement type, indicating results may have been due to providing additional calories and not the macronutrient composition (protein/carbohydrate).

Previous work indicates that IET soldiers may be training in an energy deficit [11,12,24]. This deficit may have become more serious since 2012–2013 when the Army implemented a new program to educate soldiers about healthy food selection. A study performed in 2002 indicated that soldiers were eating more than 3500 calories/day [24]. However, a study performed after the implementation of the healthy eating program found soldiers were consuming only 2600 calories/day, resulting in an average caloric deficit of 595 calories a day [12]. Multiple studies suggest training while in caloric deficit increases fatigue [7], decreases cognitive function [8,9], and decreases recovery [7], which may increase IET soldier’s risk of MSI. While energy expenditure and caloric intake were not measured for all groups throughout training, caloric expenditure and caloric intake was measured in the two supplements per day groups prior to supplementation, indicating a caloric deficit of 595 calories [12].

Training in a caloric deficit can produce weight loss. This loss comes from decreased fat mass, but also losses in fat free mass (muscle), leading to muscular damage and increased fatigue rates [7,11]. During a 8-week Army course, soldiers who trained under a caloric deficit, reported fatigue up to five-weeks following training [7]. Additionally, soldiers training in caloric deficit exhibited significantly reduced skeletal muscle metabolic and contractile function due to chronic improper recovery [7]. Creatine kinase (muscle damage biomarker) was greater following an acute bout of exercise in participants training during a one-week caloric deficit compared to no energy deficit [25]. Skeletal muscle damage and fatigue from intense, repetitive training sessions can increase a soldier’s chance of sustaining an MSI through alterations in movement patterns and an inability to recover from successive muscular micro-trauma.

Stress injuries, especially stress fractures of the femoral neck and tibia, are costly and recurrent problems within IET [26]. Caloric deficits in athletes have been shown to have a negative effect on bone formation and reabsorption [27,28]. A study examining the effect of energy deficit in runners found that blood P1NP content (osteoblast activity biomarker), was significantly decreased after training in a caloric deficit [28]. IGF-1 (bone formation biomarker), was also decreased in runners following training in a caloric deficit and in soldiers completing ranger training in a caloric deficit [28,29]. Thus, increasing caloric intake through protein/carbohydrate supplementation may have a positive impact on bone formation and stress fracture rates in IET.

Our findings of decreased MSI rates in supplemented groups may also have resulted from nutrient timing. A typical day in IET includes physical training from 0500–0700, followed by breakfast between 0700–0830 [3]. Lunch and dinner are normally served around 1200 and 1700, respectively [3]. The day ends with light/moderate activity before the soldiers begin a rest cycle from 2200–0500. In the current study, soldiers MSI rates were lower in soldiers who consumed two servings/day of either protein or carbohydrate following morning physical training and prior to bed. Past research has indicated that consuming protein 30–120 min after training promotes performance and recovery benefits derived from increased blood circulation and muscle protein synthesis [30]. Additionally, consuming protein prior to bed has been shown to increase muscle protein synthesis facilitating muscle recovery and strength [14,15]. Research also suggests consuming carbohydrates immediately following physical training rapidly replenishes glycogen stores, positively impacting the body’s capacity to deploy crucial energy reserves throughout the near eighteen-hour training day [20]. This is necessary when activity is initiated within four hours of the previous physical activity, which is common during IET training [20]. In addition to nutrient timing, our decreased MSI rates may have resulted from decreased time between meals. IET soldiers can exist in a food-deprived state for up to 13 h between dinner and breakfast. During this time, soldiers continue to complete tasks and physical training that includes vigorous morning physical training. The lack of caloric intake during this time can have an effect similar to long-term caloric deficit on fatigue rates, decreased cognitive function, and the inability of the muscles to recover from exertion [7,8,9].

Finally, it is notable that caloric deficit and weight loss have also been shown to impair cognitive function [8,9]. Participants who are dieting have been shown to have slower reaction times, poorer vigilance performance, and decreased immediate recall of words [8,9]. During IET soldiers are taught and evaluated on over 180 collective tasks, all of which required peak cognition performance. Cognitive impairments can detract from knowledge transfer/capture and contribute to incorrectly or poorly completing soldier tasks, possibly putting them at a higher risk of injury. Since IET soldier have been shown to complete soldier tasks in a caloric deficit, it is possible that decreasing the caloric deficit through supplementation may have increased a soldier cognitive performance, however measures of cognitive performance were not captured in this study.

This study has several limitations. Protein/carbohydrate supplemented were distributed by drill sergeants in each platoon and not by the research team. It is possible that soldiers missed servings without the researcher’s knowledge. However, a member of the research team checked weekly to assess for compliance with supplement distribution. Implementation of this research into IET would require a similar supplementation distribution plan, therefore we felt this was a realistic assessment of the use of supplements in IET. Second, the data from non-supplemented soldiers was accessed from historical data and these soldiers were not part of the supplementation study. However, the historical data was taken from recently trained companies within the same battalion that trained during the same time of year, with the same Certified Athletic Training team recording MSI. Analyzing these historical data in this context should minimize differences in command climate and known injury rate changes that occur across time of year. Third, there was an average difference of 3.5 years and 4.5 kg between participants who consumed one supplement versus participants who consumed two supplements. Finally, dietary recalls were not collected from the historical soldiers. This limits our ability to determine if increasing calories, absolute or relative protein intake, or absolute or relative carbohydrate intake through supplementation was associated with decreased MSI rates.

## 5. Conclusions

This investigation is the first to examine the effects of protein or carbohydrate supplementation on MSI rates, limited/missed duty rates and limited/missed training days in IET. Soldiers who consumed two supplements/day were less likely to be injured and missed less training. Decreases in MSI rates and missed training may be due to decreases in a caloric deficit and/or proper nutrient timing. Future research should examine the effects of increased caloric intake from improved food consumption in the dining facility on the rate of MSI in IET soldiers and the effect of decreasing the amount of time between meals.

## Figures and Tables

**Table 1:** Summary of participants’ descriptive statistics.

Group	Age (yrs)	Height (cm)	Weight (kg)
1 WP Serving Group (*n* = 50)	21.4 ± 3.2	172.9 ± 7.6	75.1 ± 13.1
1 CHO Serving Group (*n* = 50)	22.8 ± 3.7	174.9 ± 7.7	78.1 ± 14.8
2 WP Serving Group (*n* = 56)	18.6 ± 1.0	172.8 ± 6.0	71.7 ± 12.3
2 CHO Serving Group (*n* = 56)	18.5 ± 1.2	172.9 ± 5.4	71.0 ± 11.0
Historical Data (*n* = 1963)	NC	NC	NC

Legend: All data are presented as mean ± standard deviation. Abbreviations: years old (yrs), centimeters (cm), kilograms (kg), whey protein supplement (WP), carbohydrate supplement (CHO), data not collected (NC), * Significant (p < 0.05) difference between 1 and 2 supplements.

**Table 2:** Summary of MSI Rates and Limited/Missed Duty Rates.

Group	MSI Rates	Limited/Missed Duty Rates
1 WP serving (10 weeks)	13/50 (26%)	5/13 (38%)
1 CHO serving (10 weeks)	19/50 (38%)	10/19 (53%)
2 WP servings (15 weeks)	11/56 (19%)	5/11 (45%)
2 CHO servings (15 weeks)	10/56 (17%)	5/10 (50%)
2 WP or CHO servings (15 weeks)	32/112 (28%)	16/32 (50%)
No supplementation (15 weeks)	340/499 (68%)	270/340 (79%)
2 WP or CHO servings (10 weeks)	15/112 (13%)	8/15 (53%)
1 WP or CHO serving (10 weeks)	32/100 (32%)	15/33 (45%)
No supplementation (10 weeks)	561/1464 (38%)	295/561 (53%)

Abbreviations: Whey protein supplement (WP), carbohydrate supplement (CHO).
